# Supplementary material for: A transcription factor ensemble orchestrates bundle sheath expression in rice
Source: Nat Commun. 2025 Jul 31;16:7040. doi: 10.1038/s41467-025-62087-0 (PMC12314071; doi:10.1038/s41467-025-62087-0)
Supplement: Supplementary file 1 — Supplementary Figs. [file 41467_2025_62087_MOESM1_ESM.pdf]

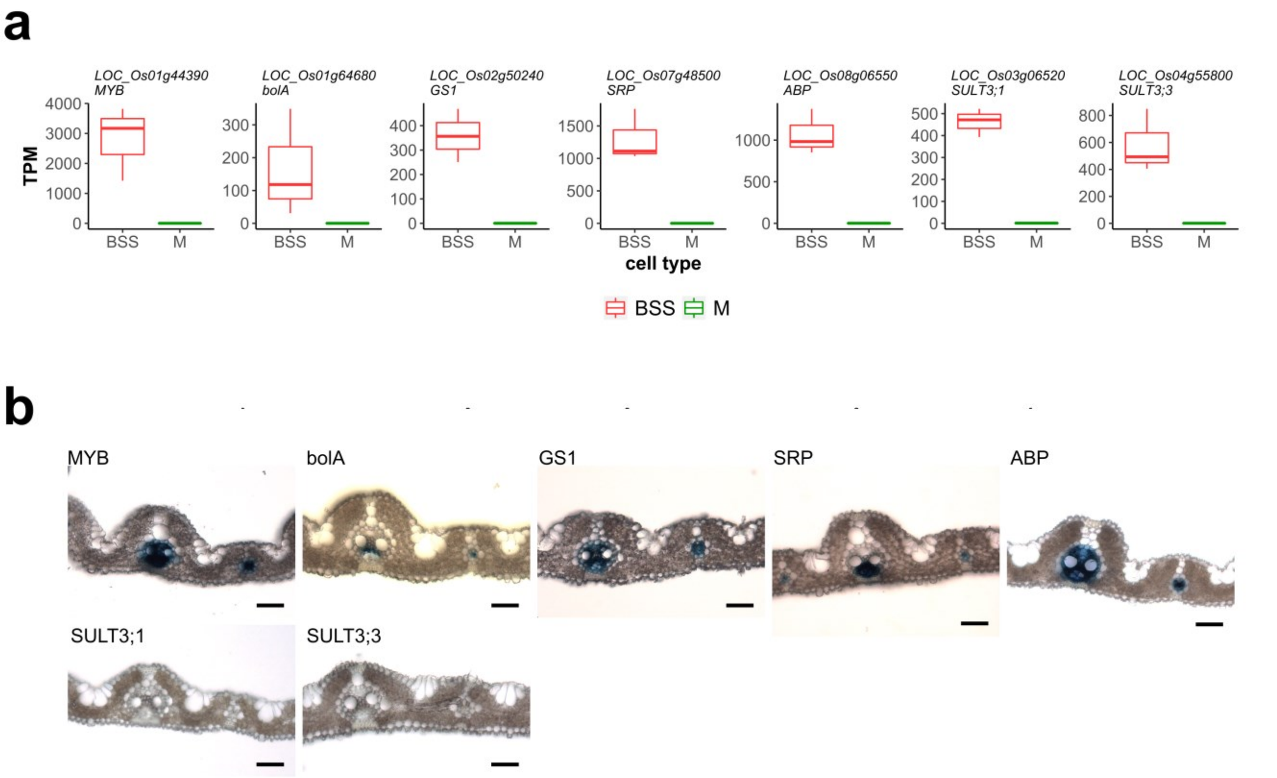

c

NO.	GENE ID	MSU7 annotation	Symbol	Region cloned	GUS localisation
1	LOC_Os01g44390	Myeloblastosis	MYB	-1641-+178	V
2	LOC_Os01g64680	Homolog of E. coli bola	bolA	-960-+345	V
3	LOC_Os02g50240	Glutamine Synthetase 1	GS1	-1976-+804	V
4	LOC_Os07g48500	Stress responsive protein	SRP	-1373-+496	V
5	LOC_Os08g06550	Acyl CoA binding protein	ABP	-1468-+805	V
6	LOC_Os03g06520	Sulfate transporter 3;1	SULT3;1	-1508-+795	No expression
7	LOC_Os04g55800	Sulfate transporter 3;3	SULT3;3	-2052-+264	No expression

Supplementary Figure 1. Analysis of seven rice promoters identified after analysis of transcripts that accumulate preferentially in bundle sheath strands. (**a**) Transcript abundance (Transcript per million, TPM) derived from each gene, box plots display the median and the 25th, 50th and 75th percentiles; whiskers extend to the furthest data points within 1.5 times of the interquartile range. (**b**) Representative GUS staining images using promoters from *MYB, bolA, GS1, SRP, ABP, SULT3;1* and *SULT3;3*. Scale bars represent 50 µm. (**c**) Summary of promoters tested including gene information, upstream flanking region (relative to translational start site), and GUS localisation. Abbreviations: BSS, Bundle Sheath Strands (vascular bundle and bundle sheath); M, Mesophyll; V, vascular bundle (cell types including xylem, phloem).

a

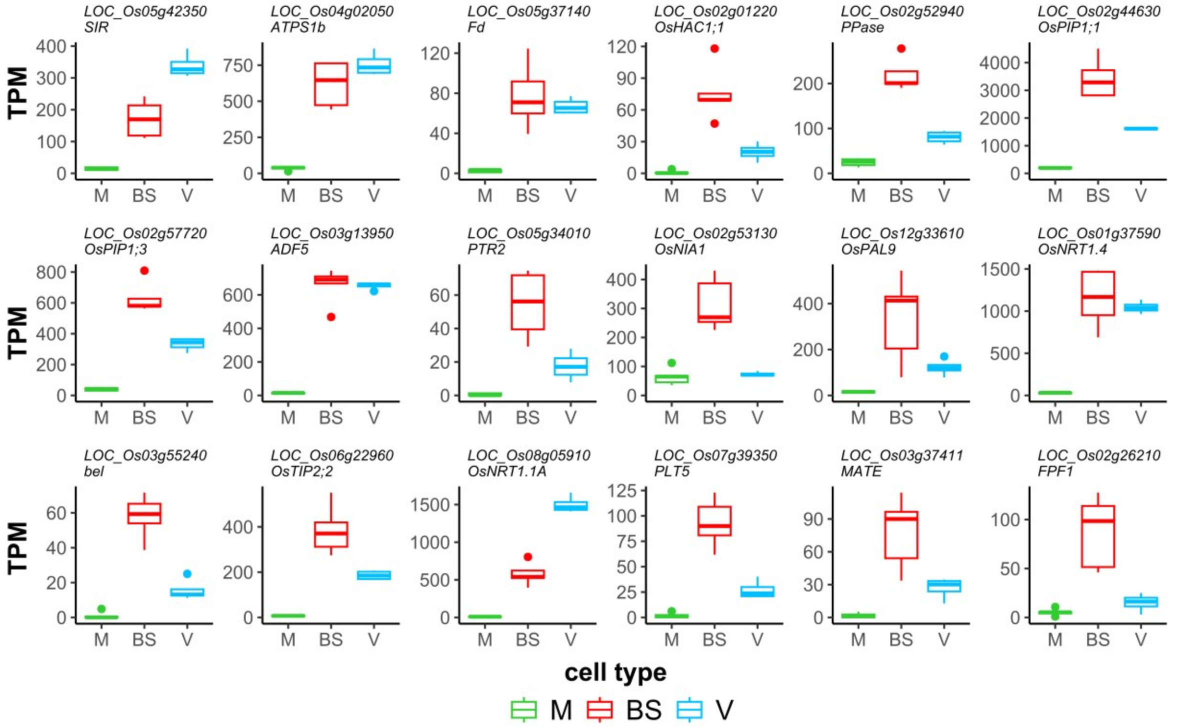

b

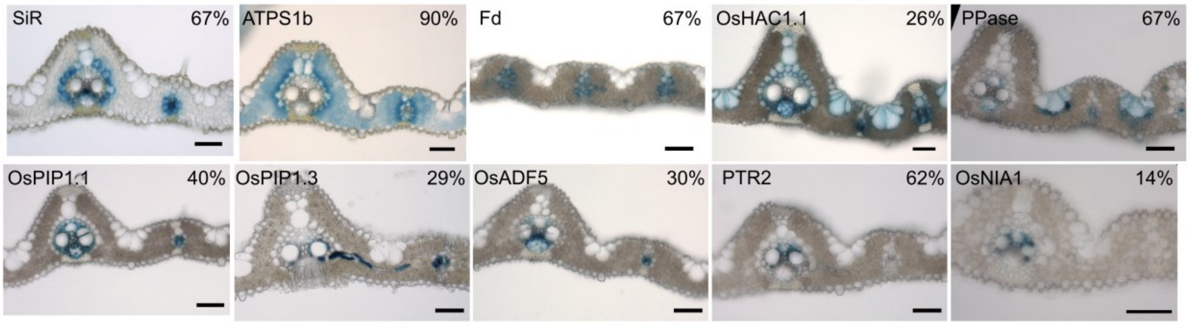

c

NO.	GENE ID	MSU7 annotation	Symbol	Biological process	Region cloned	GUS localisation
1	LOC_Os05g42350	ferredoxin--nitrite reductase	SIR	Sulfur metabolism	-2571 - +42	BS and V
2	LOC_Os04g02050	bifunctional 3-phosphoadenosine 5-phosphosulfate synthetase	ATPS1b	Sulfur metabolism	-1769 - +2081	BS and M
3	LOC_Os05g37140	2Fe-2S iron-sulfur cluster binding domain containing protein (ferredoxin)	Fd	Sulfur metabolism	-1745 - +108	BS, M and V
4	LOC_Os02g01220	rhodanese-like domain containing protein	OsHAC1;1	Sulfur metabolism	-3011 - -1	BS, V and epidermis
5	LOC_Os02g52940	soluble inorganic pyrophosphatase	PPase	Sulfur metabolism	-1456 - +9	V
6	LOC_Os02g44630	aquaporin protein	OsPIP1;1	Water transport	-3079 - -1	V
7	LOC_Os02g57720	aquaporin protein	OsPIP1;3	Water transport	-2364 - -1	V
8	LOC_Os03g13950	actin-depolymerizing factor	OsADF5	others	-2937 - +174	V
9	LOC_Os05g34010	peptide transporter PTR2	PTR2	Solute transport	-2783 - +27	V
10	LOC_Os02g53130	nitrate reductase	OsNIA1	Nitrate metabolism	-3191 - +165	V
11	LOC_Os12g33610	phenylalanine ammonia-lyase	OsPAL9	others	-2631 - +117	No expression
12	LOC_Os01g37590	peptide transporter PTR2	OsNRT1.4	Nitrate metabolism	-2576 - +147	No expression
13	LOC_Os03g55240	cytochrome P450	bel	others	-2700 - +210	No expression
14	LOC_Os06g22960	aquaporin protein	OsTIP2;2	Water transport	-2984 - -1	No expression
15	LOC_Os08g05910	peptide transporter PTR2	OsNRT1.1A	Nitrate metabolism	-1818 - +249	No expression
16	LOC_Os07g39350	transporter family protein	PLT5	Solute transport	-2520 - +87	No expression
17	LOC_Os03g37411	MATE efflux family protein	MATE	Solute transport	-2904 - +198	No expression
18	LOC_Os02g26210	flowering promoting factor-like 1	FPF1	others	-2464 - +162	No expression

Supplementary Figure 2. Identification of bundle sheath specific promoters after analysis of transcripts that accumulate preferentially in bundle sheath compared with mesophyll cells. (**a**) Transcript abundance (Transcript per million, TPM) of eighteen candidate genes in bundle sheath (BS), mesophyll (M) and vascular bundle (V) cells, box plots display the median and the 25th, 50th and 75th percentiles; whiskers extend to the furthest data points within 1.5 times of the interquartile range. (**b**) Representative cross sections of leaves after GUS staining showing bundle sheath or vascular bundle expression. Percentage of transgenic lines with each GUS pattern indicated upper-right, in the remaining lines GUS was not detectable. Scale bars represent 50 µm. (**c**) Summary of gene information, biological process, region cloned (relative to translational start site) and GUS localisation. Abbreviations: BS, Bundle Sheath; M, Mesophyll; V, Vascular bundle (cell types including xylem, phloem which are surrounded by bundle sheath cells).

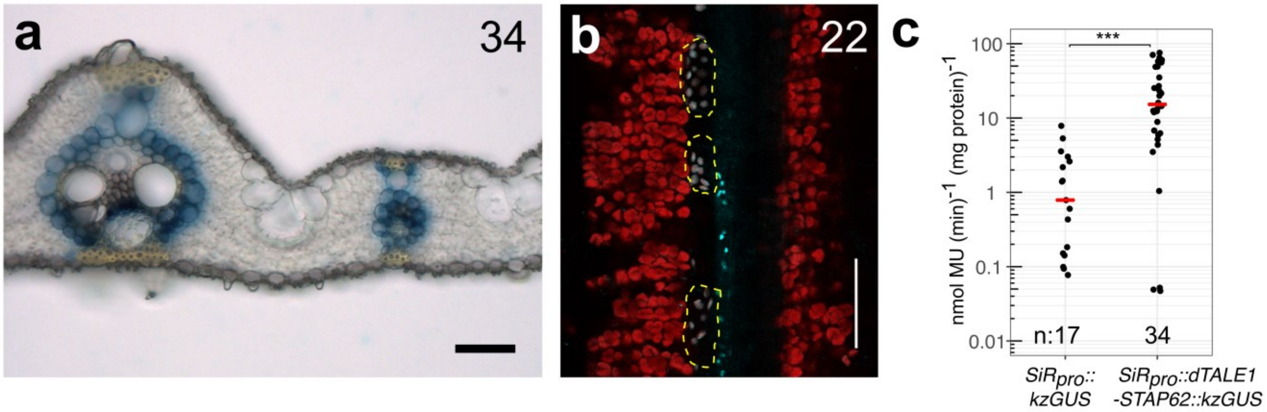

Supplementary Figure 3. The domesticated *SiR* promoter combined with the dTALE/STAP system drives strong expression in the rice bundle sheath. (**a**) Representative image of expression from the domesticated *SiR* promoter driving dTALE and STAP4-GUS in transverse section of rice leaf, number of replicates were indicated in top-right corner. (**b**) Representative image of expression from of nuclear localized mTurquoise2 fluorescent protein under control of the domesticated *SiR* promoter driving dTALE, and mTurquoise2 downstream of *STAP62*. Bundle sheath cells marked by yellow dashed lines, and red indicates chlorophyll autofluorescence, number of replicates were indicated in top-right corner. (**c**) GUS activity mediated by dTALE and *STAP62* compared with the native *SiR* promoter. Data were subjected to a two-sided Wilcoxon rank-sum test, *** represents $P < 0.001$. Median catalytic rate of GUS were indicated in red line, number of T_0 transgenic plants analyzed were shown at the bottom of plot. Source data are provided as a Source Data file.

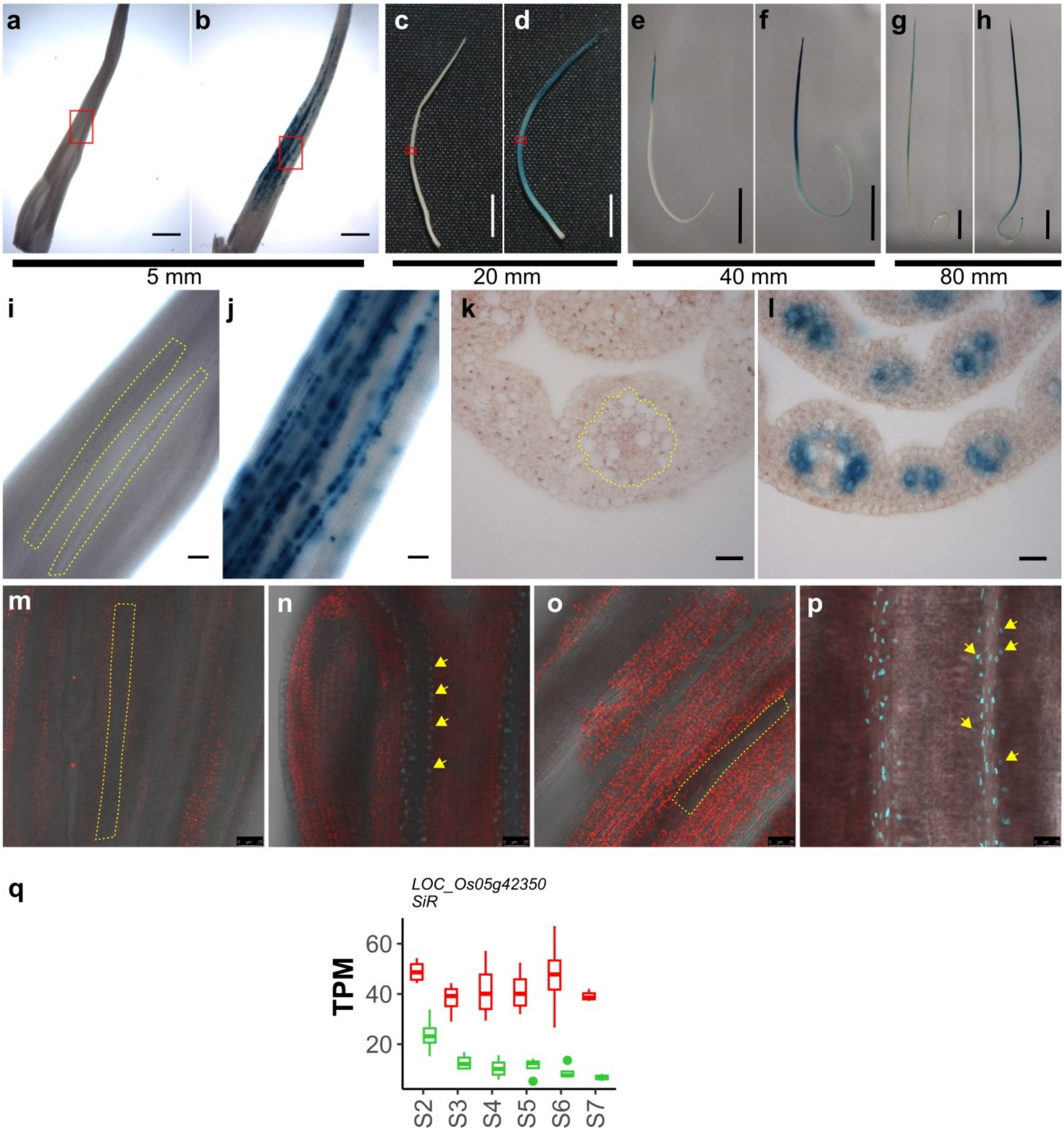

Supplementary Figure 4. The rice *SiR* promoter drives expression in bundle sheath cells earlier than the *Zoysia japonica PCK* promoter. GUS in transgenic rice plants expressing *ZjPCKpro::kzGUS* (**a, c, e, g**) and *SiRpro::kzGUS* (**b, d, f, h**) from 5-mm (**a, b**) 2-cm (**c, d**), 4-cm (**e, f**) and 8-cm (**g, h**) fourth leaves. **i&j** show magnified views of the boxed regions in **a** and **b**. **k&l** show leaf cross sections of the boxed regions of **c&d**. Expression of green fluorescent protein (**c, i**) and mTurquoise2 (**f, l**) in transgenic plants expressing *ZjPCKpro::dTALE-STAP4::GFP-NLS* (**m, o**) and *SiRpro::H2B-mTurquoise2* (**n, p**) from 5-mm and 2-cm fourth leaves. NLS and H2B are nuclear localisation signal peptides. Yellow arrows indicate nuclei of bundle sheath cells expressing H2B-mTurquoise2, bundle sheath strands in *ZjPCKpro* lines were highlighted with yellow dash lines. Chlorophyll autofluorescence indicated in red (**m-p**). **(q)** Transcript per million (TPM) of *SiR* in bundle sheath strands (BSS) and mesophyll (M) cells during leaf maturation, box plots display the median and the 25th, 50th and 75th percentiles; whiskers extend to the furthest data points within 1.5 times the interquartile range. Leaf developmental stage S2 to S7 represent base of the 4th leaf at the 6th, 8th, 9th, 10th, 13th and 17th day after sowing. Scale bars represent 500 µm for **a,b**; 5 mm for **c-h**; 50 µm for **i-l**; 25 µm for **m-p**.

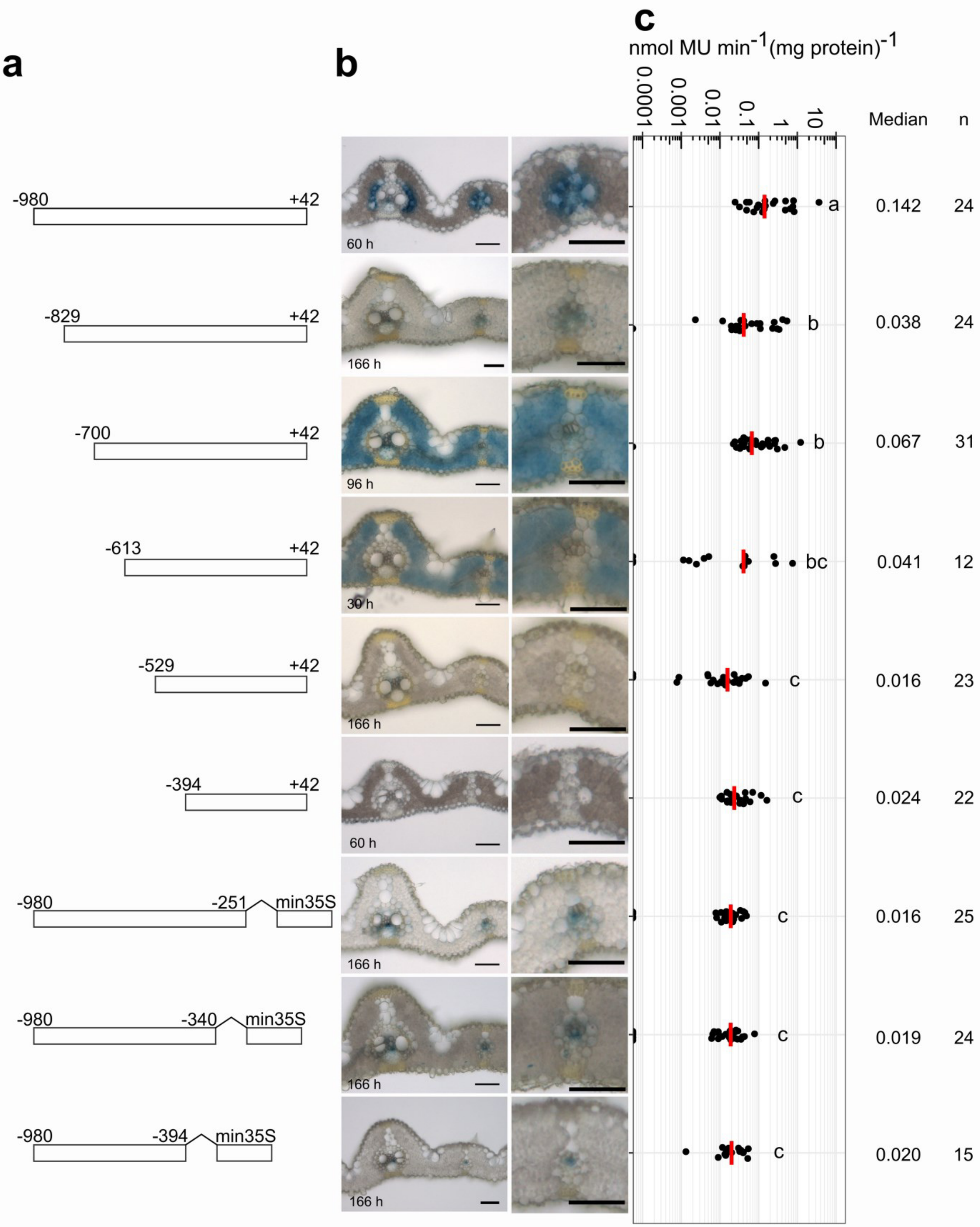

Supplementary Figure 5. Impact of 5' and 3' deletions on patterning of GUS from the *SiR* promoter. (**a**) Schematics showing sequences fused to GUS reporter. (**b**) Representative images of leaf cross sections after GUS staining. (**c**) Promoter activity determined by the fluorometric 4-methylumbelliferyl-β-D-glucuronide (MUG) assay. Data subjected to pairwise two-sided Wilcoxon rank-sum test with Benjamini-Hochberg correction. Lines with differences in activity that were statistically significant (adjusted *P*<0.05) labelled with different letters. Median catalytic rate of GUS indicated with red lines, n indicates total number of T_0 transgenic plants assessed. Source data are provided as a Source Data file.

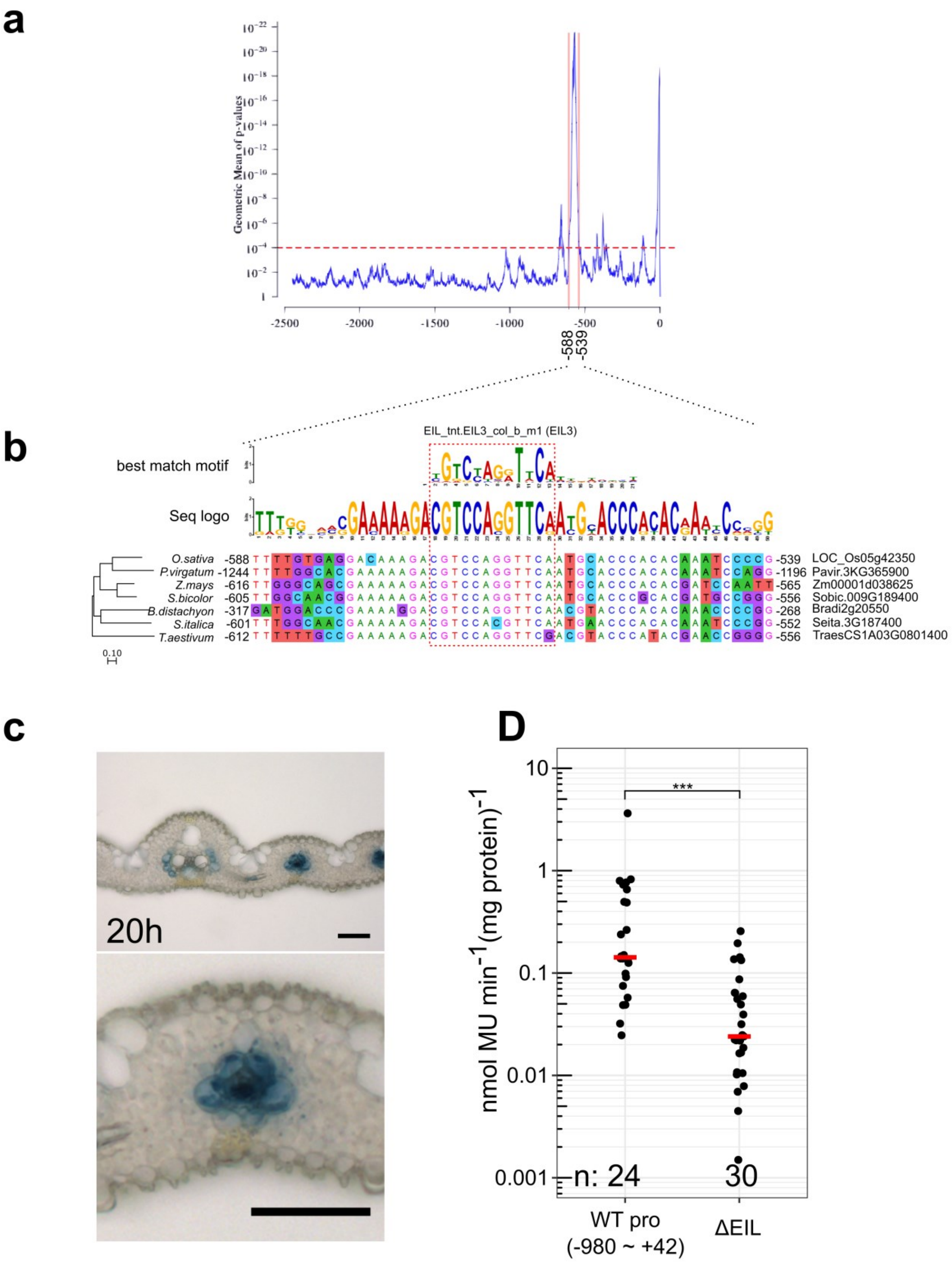

Supplementary Figure 6. The evolutionally conserved *ETHYLENE INSENSITIVE3-LIKE* (*EIL*) binding site regulates expression level but not cell specificity of the *SiR* promoter. (**a**) Conservation profile of 50-bp sliding window of the *SiR* promoter from seven grass species using EARS tool[2]. (**b**) Sequence alignment at region -588 to -539 bp and the best match motif for this region. (**c**) Representative image of leaf cross sections after GUS staining in T_0 transgenics transformed with *SiR* promoter (nucleotides -980 to +42) with *EIL* binding site deleted. (**d**) Promoter activity determined by the fluorometric 4-methylumbelliferyl-β-D-glucuronide (MUG) assay. Data were subjected to a Wilcoxon rank-sum test, *** represents *P <0.001,* median catalytic rate of GUS indicated with the red line, number of replicates shown at the bottom of plot. Source data are provided as a Source Data file.

.

a

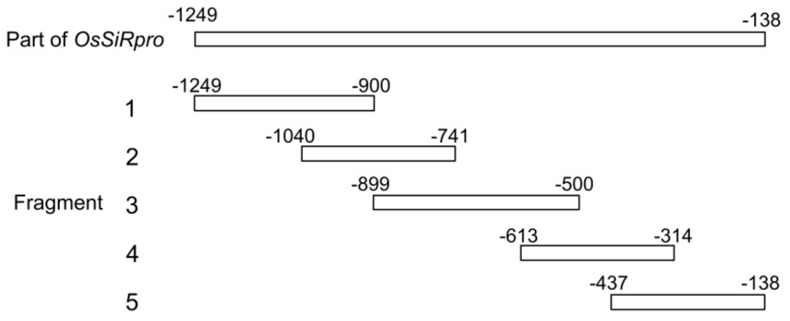

b

Fragment	nucleotides	confidence	MSU ID	Symbol	TF family
1	-1249 .. -900	D	LOC_Os07g05720	OsTCP21	TCP
1	-1249 .. -900	D	LOC_Os12g37410	OsOBF1,OsbZIP87	bZIP_A/Ocs
2	-1040 .. -741	D	LOC_Os12g37410	OsOBF1,OsbZIP87	bZIP_A/Ocs
2	-1040.. -741	A	LOC_Os02g54160	EREBP1	ERF/DREB_A
2	-1040.. -741	D	LOC_Os03g64260	OsERF83	ERF/DREB_A
2	-1040.. -741	A	LOC_Os04g52090	OsAP2-39	ERF/DREB_A
2	-1040.. -741	A	LOC_Os01g58420	AP37,OsERF3	ERF/DREB_A
2	-1040.. -741	A	LOC_Os05g41780	OsERF74	ERF/DREB_A
2	-1040.. -741	D	LOC_Os09g26420	OsERF72	ERF/DREB_A
3	-899 .. -500	D	LOC_Os04g52090	OsAP2-39	ERF/DREB_A
3	-899 .. -500	A	LOC_Os03g20780	OsEIL1	EIL
3	-899 .. -500	A	LOC_Os07g48630	OsEIL2	EIL
3	-899 .. -500	D	LOC_Os09g31400	OsEIL3	EIL
4	-613 .. -314	A	LOC_Os01g69980	TCP20	TCP
4	-613 .. -314	D	LOC_Os07g43420	OsFLP	MYB_C
4	-613 .. -314	B	LOC_Os08g43160	PCF2,OsPCF2	TCP
5	-437 .. -138	D	LOC_Os01g55750	OsTCP5	TCP
5	-437 .. -138	A	LOC_Os01g69980	TCP20	TCP
5	-437 .. -138	D	LOC_Os03g21060	NAC58, OsNAP	NAC_B
5	-437 .. -138	D	LOC_Os07g05720	OsTCP21	TCP
5	-437 .. -138	A	LOC_Os08g43160	PCF2,OsPCF2	TCP

c

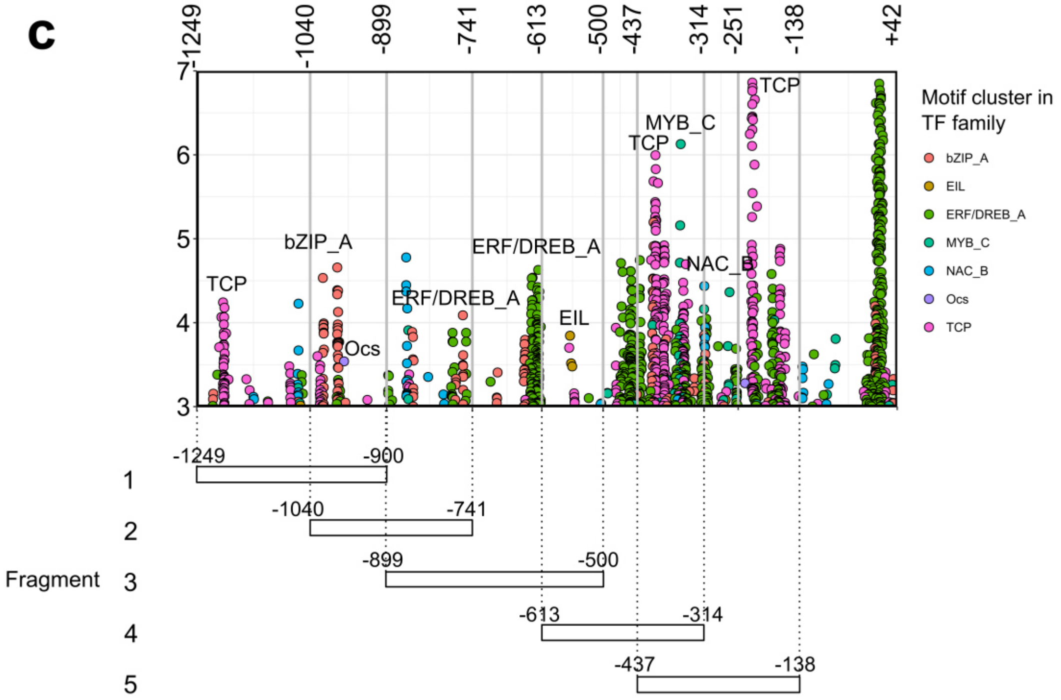

d

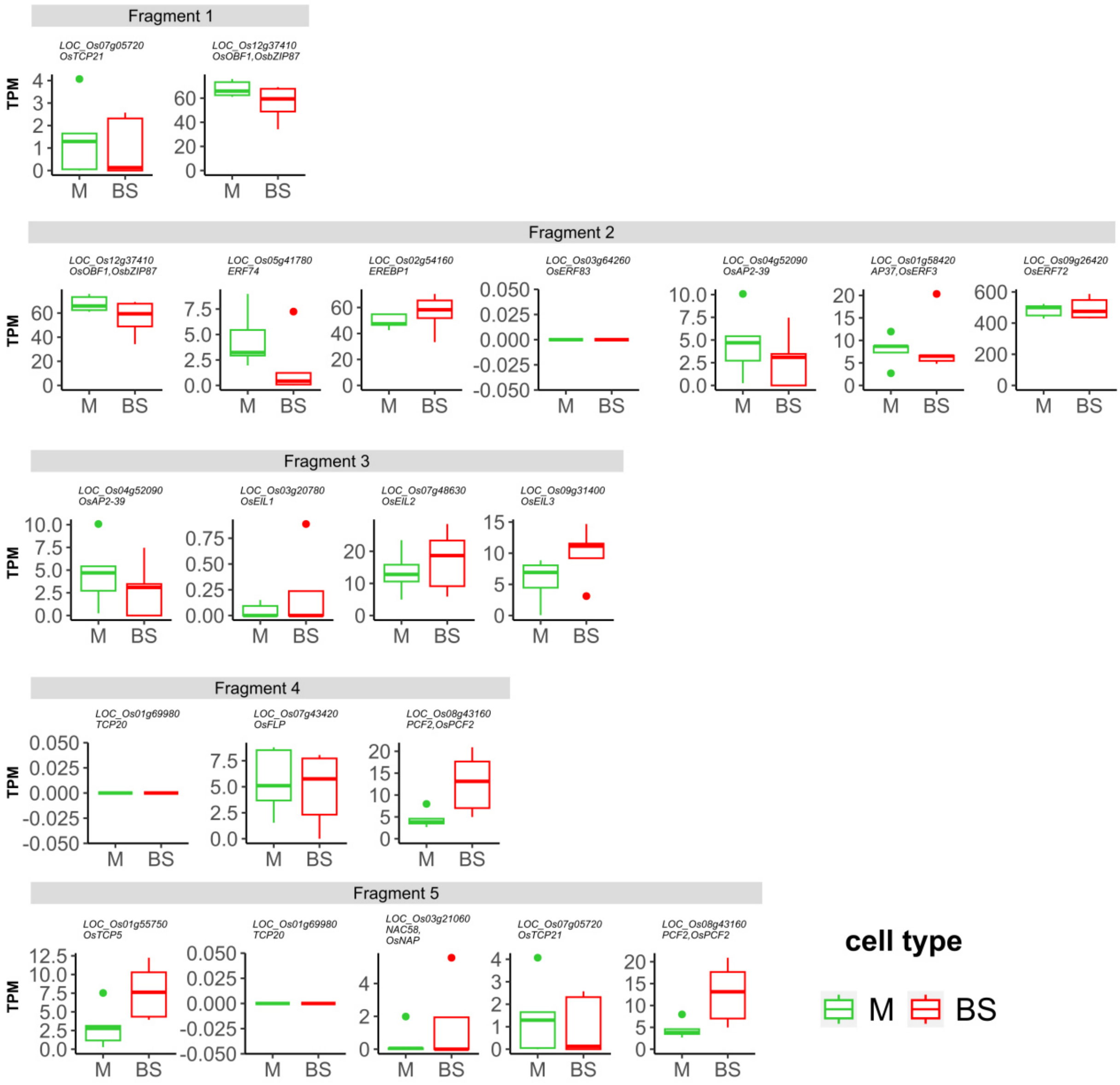

Supplementary Figure 7. Identification of transcription factors interacting with the *SiR* promoter using yeast one hybrid. (**a**) Schematic showing five fragments covering region of the *SiR* promoter used for yeast one hybrid analysis. (**b**) Transcription factors identified as interacting with each fragment. (**c**) Predicted binding sites of transcription factors in each fragment, -$\log_{10}$ transformed *p* values which was calculated from the log-likelihood score by the FIMO program (**d**) Transcript abundance for transcription factor genes in bundle sheath and mesophyll cells[1], box plots display the median and the 25th, 50th and 75th percentiles; whiskers extend to the furthest data points within 1.5 times the interquartile range.

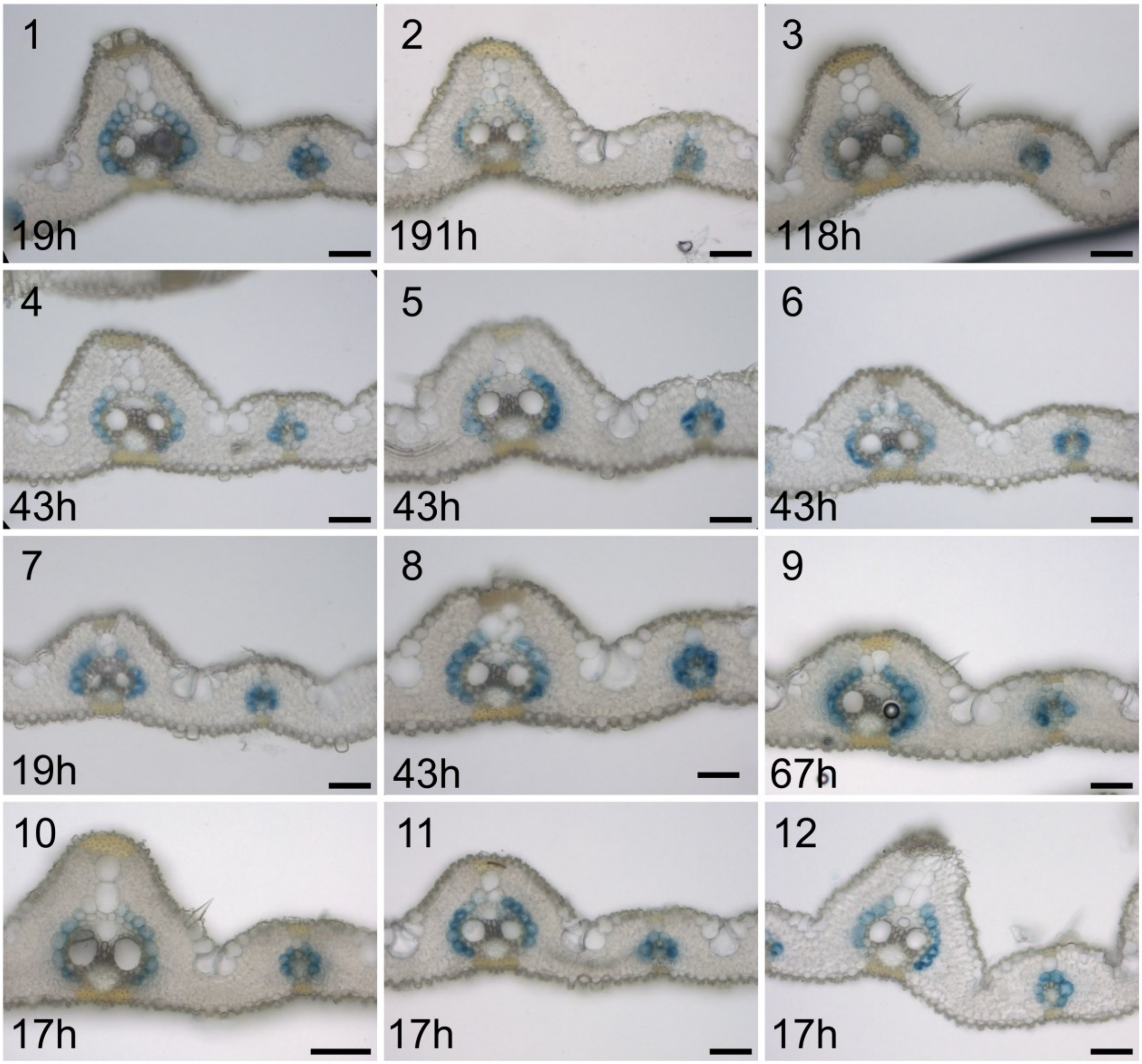

Supplementary Figure 8. Nucleotides -980 to -829 in combination with nucleotides -251 to +42 produce bundle sheath specific expression. Twelve independent lines assessed. The staining duration is displayed in the bottom-left corner, scale bars = 50 µm.

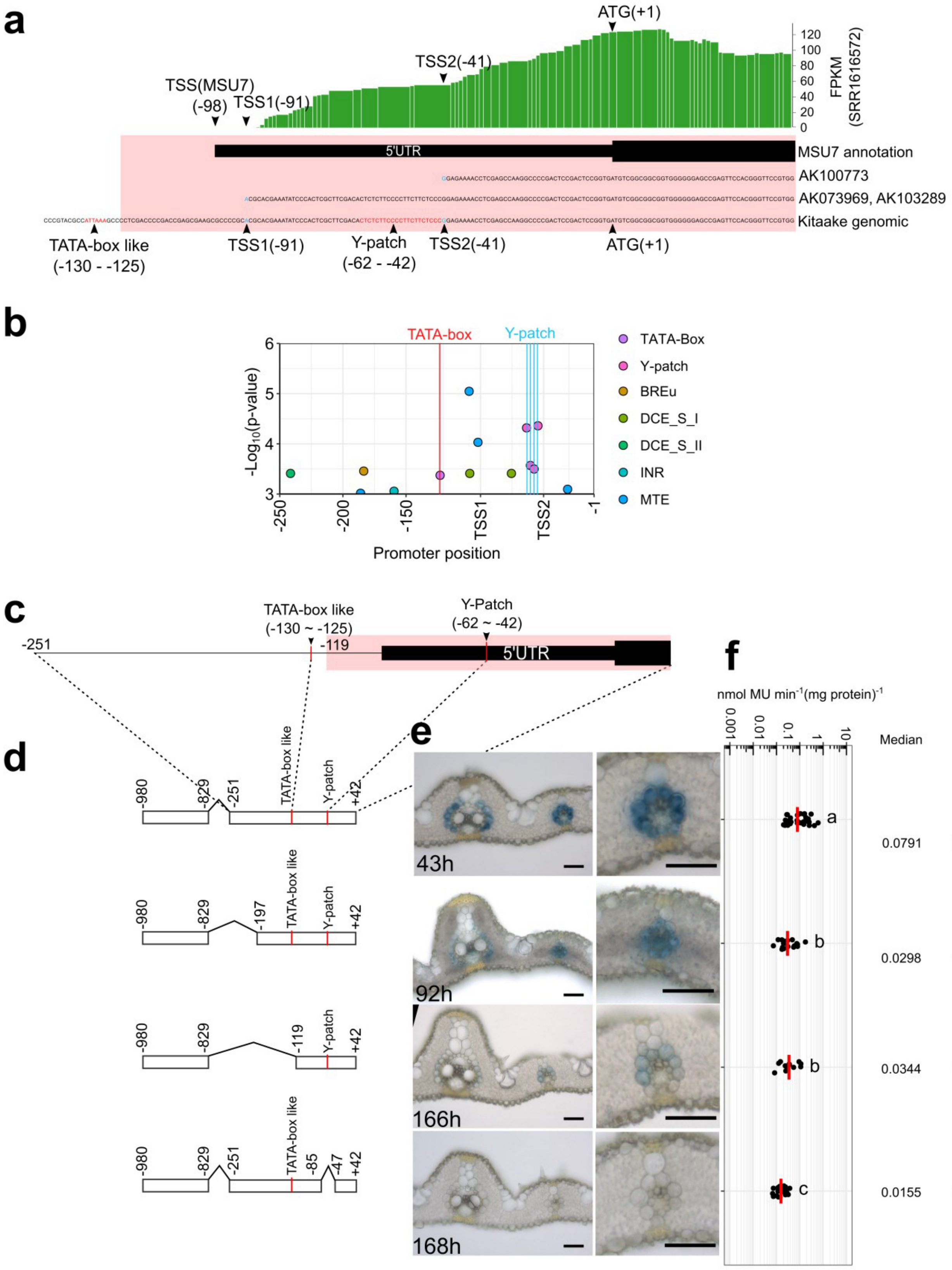
a
ATG(+1)
TSS2(-41)
TSS(MSU7)
(-98)
TSS1(-91)
FPKM
(SRR1616572)
5'UTR
MSU7 annotation
AK100773
AK073969, AK103289
Kitaake genomic
TATA-box like
(-130 - -125)
TSS1(-91)
Y-patch
(-62 - -42)
TSS2(-41)
ATG(+1)
b
TATA-box
Y-patch
-Log10(p-value)
Promoter position
TSS1
TSS2
TATA-Box
Y-patch
BREu
DCE_S_I
DCE_S_II
INR
MTE
c
TATA-box like
(-130 ~ -125)
Y-Patch
(-62 ~ -42)
-251
-119
5'UTR
d
e
f
nmol MU min-1(mg protein)-1
Median
n
43h
92h
166h
168h
0.0791
29
0.0298
17
0.0344
13
0.0155
21

Supplementary Figure 9. Nucleotides -251 to -1 serve as the core promoter. (**a**) Two transcription start sites are supported by full length cDNA clones (TSS1: AK073969, AK103289; TSS2: AK100773) and leaf RNA sequencing reads (SRR1616572). Relative position to TATA box variant (TATA var. -130 to -125bp) and Y-Patch core promoter element (-62 to -42bp) depicted, and frequency of leaf RNAseq reads indicated with green bars. (**b**) Profile of general transcription factor binding sites associated with RNA Polymerase II, $-\log_{10}$ transformed *p* values which was calculated from the log-likelihood score by the FIMO program. (**c, d**) Diagrams showing position of core promoter elements TATA box and Y-patch between nucleotides -251 to +42 (**c**), and sequences fused to GUS (**d**). (**e**) Representative images of leaf cross sections after GUS staining with zoomed-in images of lateral veins shown in right panels, scale bars = 50 um. (**f**) Promoter activity determined by the fluorometric 4- methylumbelliferyl-β-D-glucuronide (MUG) assay. n indicates total number of transgenic lines assessed. Data subjected to a pairwise two-sided Wilcoxon rank-sum test with Benjamini-Hochberg correction. Lines with differences in activity that were statistically significant (adjusted $P<0.05$) labelled with different letters. Median catalytic rate of GUS indicated with red line, n indicates total number of T_0 transgenic plants assessed. Source data are provided as a Source Data file. Abbreviations of core promoter elements in **b**: INR, initiator; MTE, motif ten element; BREu, TFIIB recognition element upstream; DCE_S_I, downstream core element S-I; DCE_S_II, downstream core element S-II.

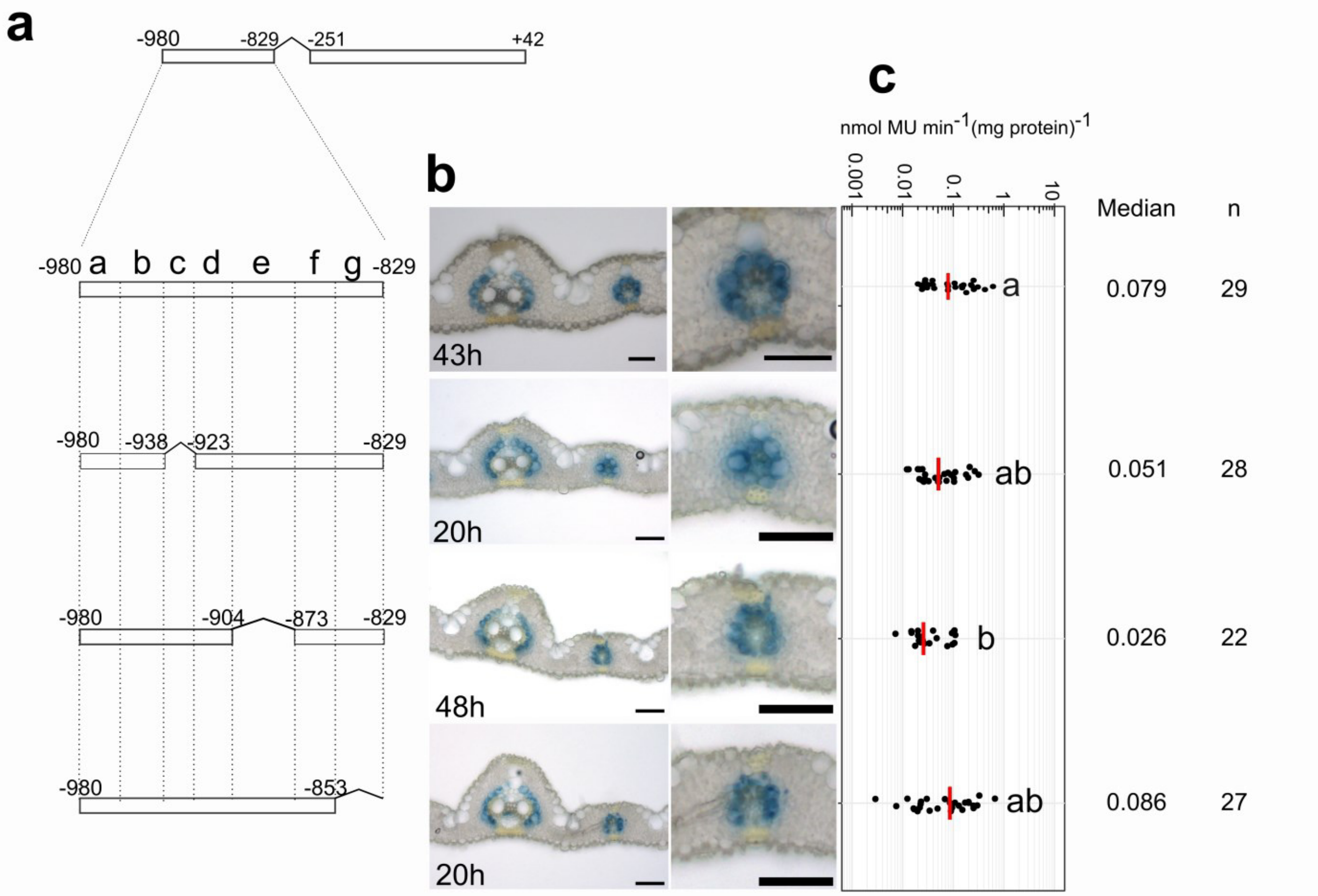

Supplementary Figure 10. Subregions in distal CRM not required for bundle sheath specific expression between nucleotides -980 and -829. (**a**) Schematic of sequences fused to GUS reporter. (**b**) Representative images of leaf cross sections after GUS staining, zoomed-in images of lateral veins shown in right panels, scale bars = 50 um. (**c**) Promoter activity determined by the fluorometric 4-methylumbelliferyl-β-D-glucuronide (MUG) assay. Data subjected to pairwise two-sided Wilcoxon rank-sum test with Benjamini-Hochberg correction. Lines with differences in activity that were statistically significant (adjusted *P*<0.05) labelled with different letters. Median catalytic rate of GUS indicated with red line, n indicates total number of T_0 transgenic plants assessed. Source data are provided as a Source Data file.

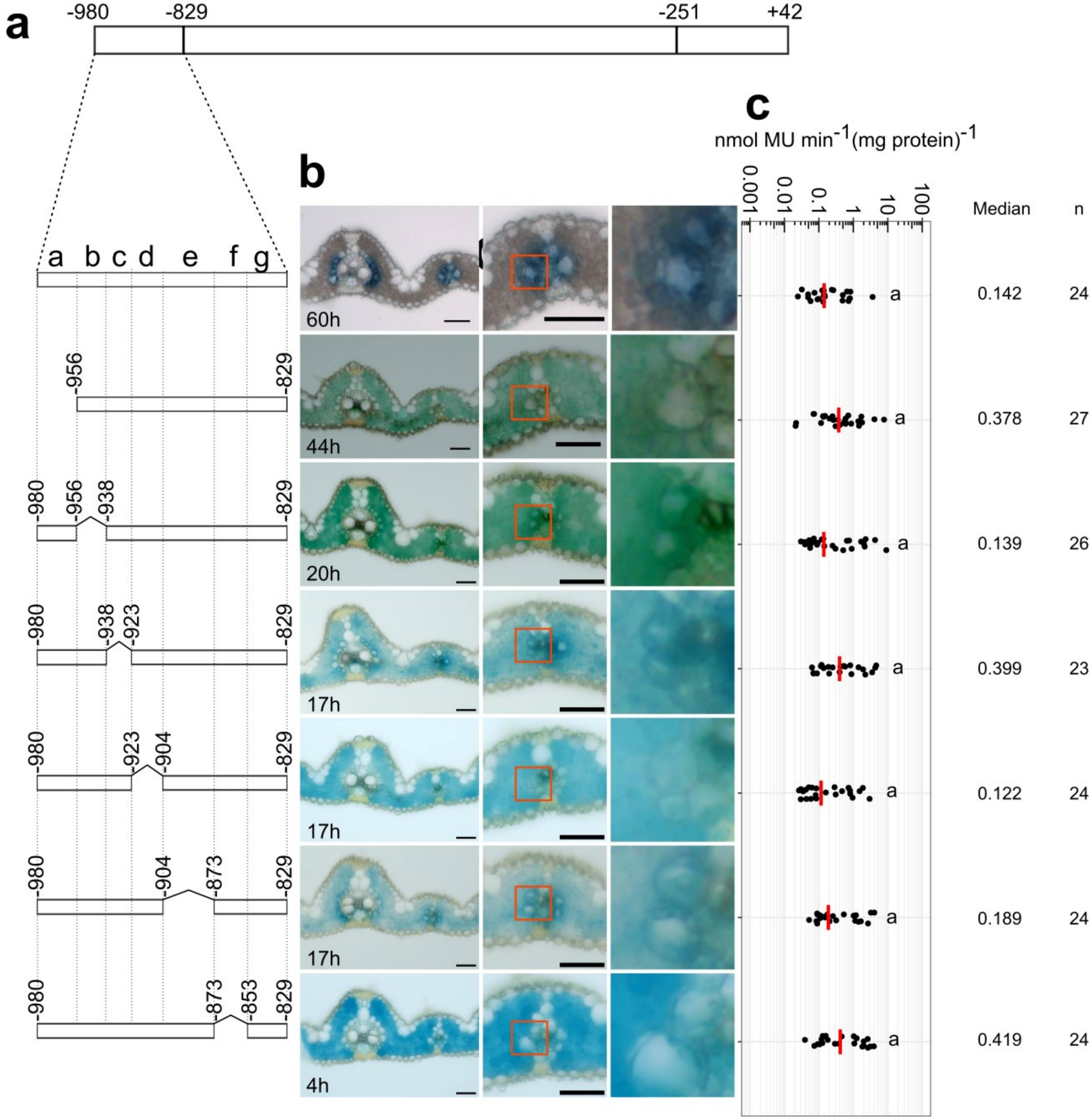

Supplementary Figure 11. Regions between nucleotides -980 and -829 combined with nucleotides -828 to -252 repress mesophyll expression. (**a**) Diagram showing deletions between nucleotides -980 and -829. (**b**) Representative images of leaf cross sections after GUS staining, zoomed-in images of lateral veins and bundle sheath cells shown in right panels, scale bars = 50 µm. (**c**) Promoter activity determined by the fluorometric 4-methylumbelliferyl-β-D-glucuronide (MUG) assay, Data subjected to pairwise two-sided Wilcoxon rank-sum test with Benjamini-Hochberg correction. Lines with differences in activity that were statistically significant (adjusted *P*<0.05) labelled with different letters. Median catalytic rate of GUS indicated with red lines, n indicates total number of T_0 transgenic plants assessed. Source data are provided as a Source Data file.

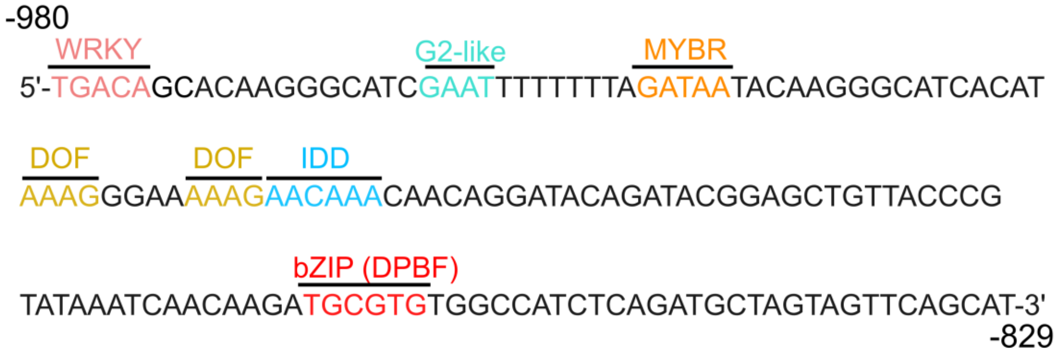

Supplementary Figure 12. Nucleotide sequence of the distal CRM. FIMO predicted transcription factor binding sites for WRKY, G2-like, MYBR, DOF, IDD, and bZIP are highlighted.

Targeted sub-region	Family	TF Symbol	MUS7 ID	Motif analysis	BS or BSV[1]	Module 15&17 (leaf developmental gradient)	M0047[2]	Ortholog of JASPAR TFs	highly similar paralogues to JASPAR TFs
a	WRKY	WRKY1	LOC_Os01g14440	FIMO			Yes		
a	WRKY	WRKY94	LOC_Os12g40570	FIMO				Yes	Yes
a	WRKY	WRKY121	LOC_Os03g53050	FIMO	Yes	Yes		Yes	
a	G2like	GLK1	LOC_Os06g24070	FIMO				Yes	
a	G2like	GLK2	LOC_Os01g13740	FIMO				Yes	
a	G2like	MYS1	LOC_Os12g01490	FIMO				Yes	
a	G2like	DLN168	LOC_Os06g35140	FIMO				Yes	
a	G2like	MYR2	LOC_Os03g03760	FIMO				Yes	
a	G2like	DLN52	LOC_Os02g14490	FIMO				Yes	
a	G2like	HINGE1	LOC_Os04g56990	FIMO	Yes				
a	G2like	DLN210	LOC_Os08g33750	FIMO	Yes	Yes			
a	G2like	Os2R_MYB2	LOC_Os01g04930	FIMO		Yes			
a	G2like	Os2R_MYB57	LOC_Os05g37730	FIMO	Yes				
a	G2like	UCIP5	LOC_Os12g39640	FIMO	Yes	Yes			
a	MYBR	OsMYBS1	LOC_Os01g34060	FIMO				Yes	
a	MYBR	OsMYBS2	LOC_Os10g41260	FIMO	Yes	Yes		Yes	
a	MYBR	OsMYBS3	LOC_Os10g41200	FIMO					Yes
a	MYBR	MYBR2	LOC_Os08g04840	FIMO				Yes	
a	MYBR	MYBR3	LOC_Os06g01670	FIMO				Yes	
a	MYBR	MYBR1	LOC_Os01g09280	FIMO				Yes	
a	MYBR	MYBY2	LOC_Os03g31230	FIMO				Yes	
a	MYBR	LHY	LOC_Os08g06110	FIMO				Yes	
d	IDD	IDD1	LOC_Os03g10140	FIMO				Yes	Yes
d	IDD	IDD2	LOC_Os01g09850	FIMO	Yes	Yes			
d	IDD	IDD3	LOC_Os09g38340	FIMO				Yes	Yes
d	IDD	IDD4	LOC_Os02g45054	FIMO				Yes	Yes
d	IDD	IDD5	LOC_Os07g39310	FIMO				Yes	Yes
d	IDD	IDD6	LOC_Os08g44050	FIMO				Yes	Yes
d	IDD	IDD10	LOC_Os04g47860	FIMO				Yes	Yes
d	IDD	IDD11	LOC_Os01g39110	FIMO				Yes	Yes
f	SNAC	SNAC3	LOC_Os01g09550	FIMO	Yes	Yes		Yes	
f	SNAC	NAC3	LOC_Os07g12340	FIMO			Yes	Yes	
f	SNAC	NAC4	LOC_Os01g60020	FIMO		Yes		Yes	
f	SNAC	NAC5	LOC_Os11g08210	FIMO	Yes		Yes		
f	SNAC	NAC6	LOC_Os01g66120	FIMO			Yes		
f	SNAC	NAC9	LOC_Os03g60080	FIMO			Yes		
f	SNAC	NAP	LOC_Os03g21060	FIMO				Yes	
f	bZIP-A	bZIP9	LOC_Os09g28310	Kim et al., 2002		Yes			
f	bZIP-A	bZIP10	LOC_Os08g36790	Kim et al., 2002					Yes
f	bZIP-A	bZIP11	LOC_Os02g52780	Kim et al., 2002					Yes
f	bZIP-A	bZIP3	LOC_Os01g59760	Kim et al., 2002					Yes
f	bZIP-A	bZIP4	LOC_Os05g41070	Kim et al., 2002					Yes

Supplementary Figure 13. Transcription factors used in effector assays and selection criteria. BS&BSV[1], Module 15&17, M0047[3] represent co-expression clusters identified as being co-expressed with *SiR* across publicly available transcriptomic datasets. "JASPAR TFs" refer to transcription factors with experimentally validated DNA-binding motifs curated in the JASPAR database[4].

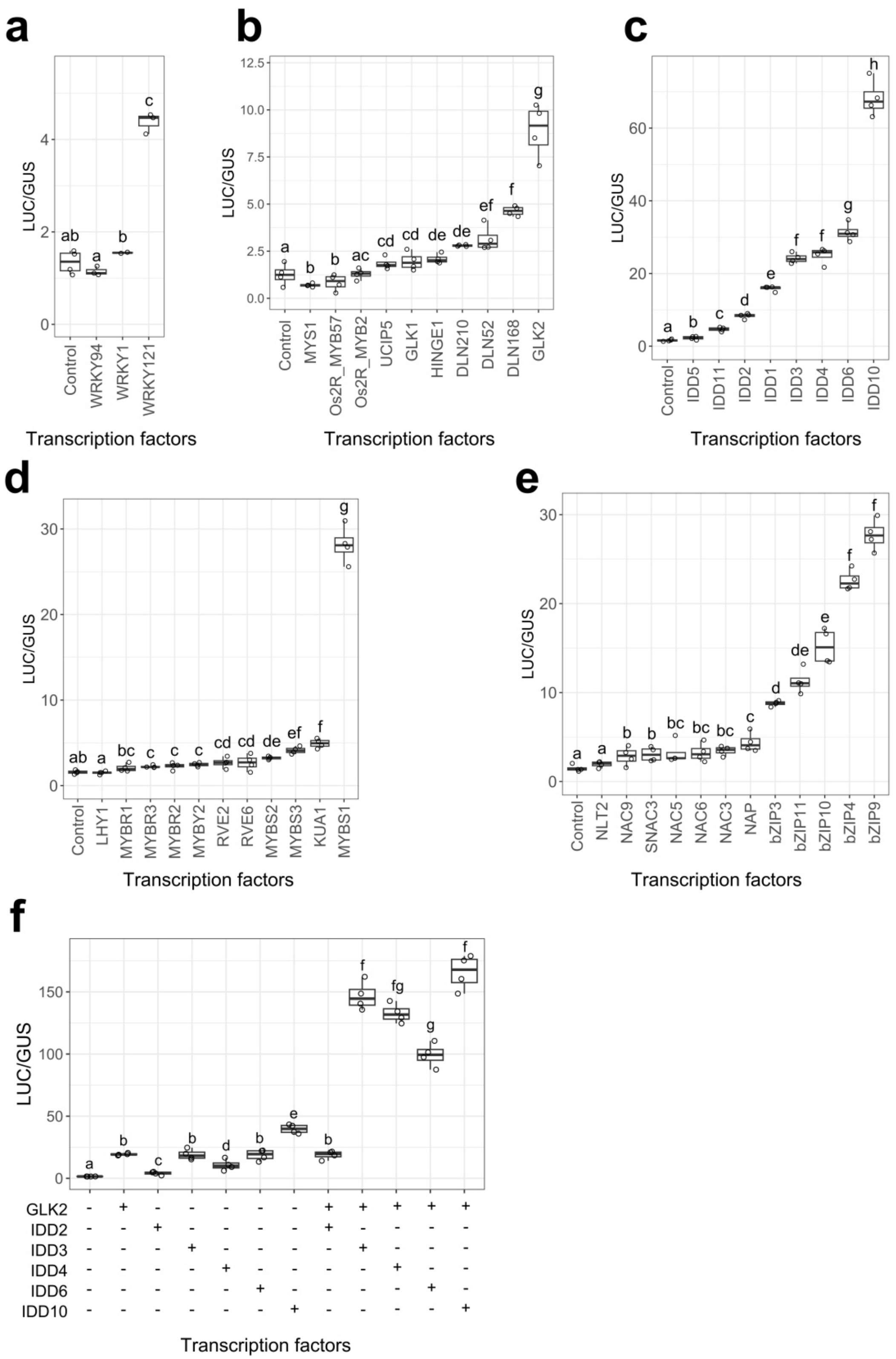

Supplementary Figure 14. Effector assay showing the effect of WRKY (a), G2-like (b), IDD (c), MYB-related (d), SNAC and bZIP (DPBF) (e) transcription factors and co-expression of GLK2 and IDDs (f) on outputs from the distal CRM. Log_2 transformed LUC/GUS ratio is subjected to pairwise t-test with Benjamini-Hochberg correction, box plots display the median and the 25th, 50th and 75th percentiles; whiskers extend to the furthest data points within 1.5 times the interquartile range, samples with significantly different activities (adjusted $P<0.05$) are labelled with different letters. Source data are provided as a Source Data file.

a

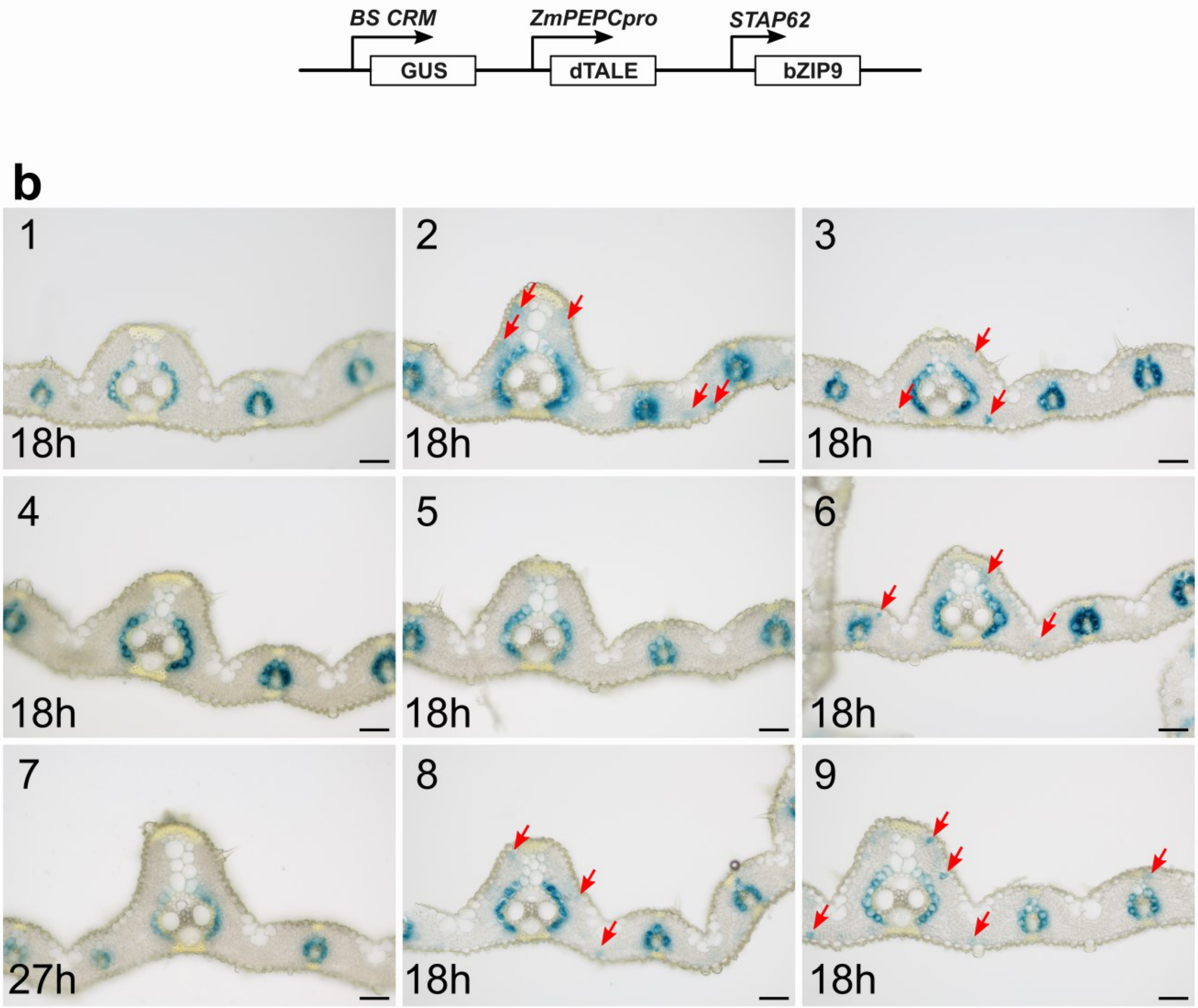
BS CRM
ZmPEPCpro
STAP62
GUS
dTALE
bZIP9
b
1
2
3
4
5
6
7
8
9
18h
18h
18h
18h
18h
18h
27h
18h
18h

c

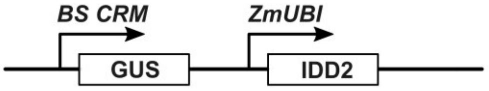

d

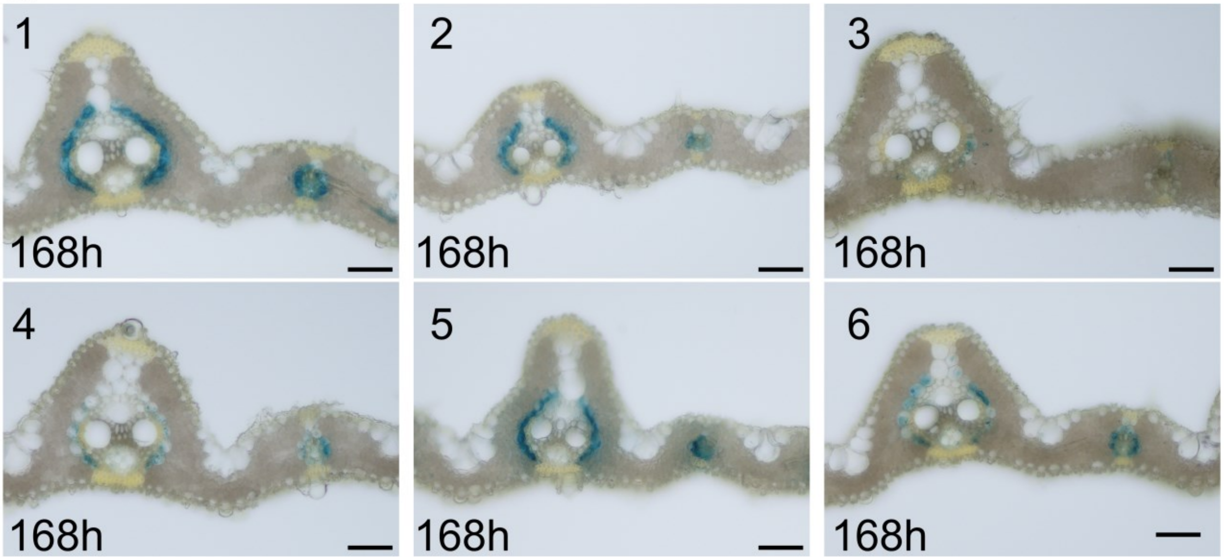

e

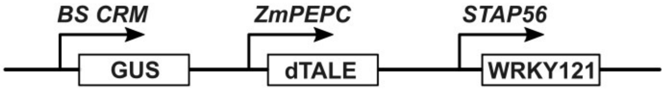

BS CRM
ZmPEPC
STAP56
GUS
dTALE
WRKY121

f

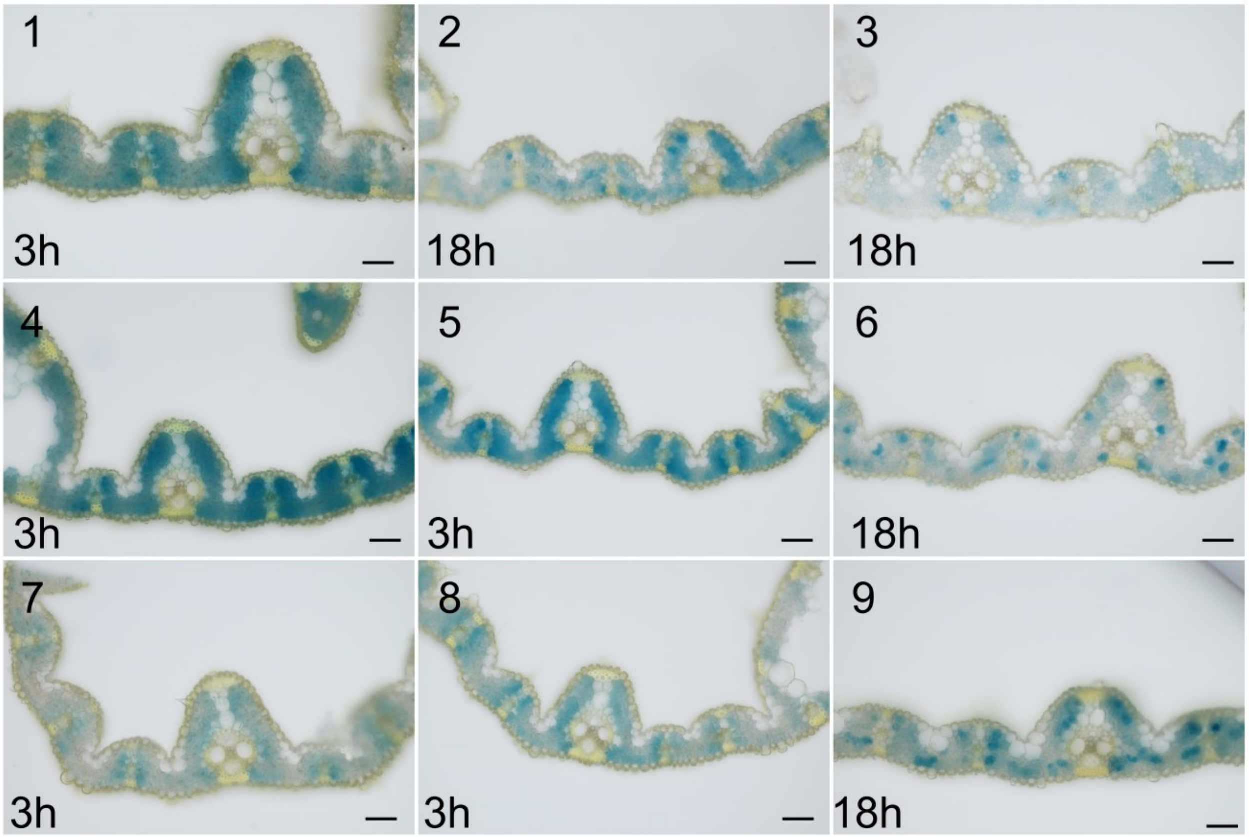

1
3h
2
18h
3
18h
4
3h
5
3h
6
18h
7
3h
8
3h
9
18h

g

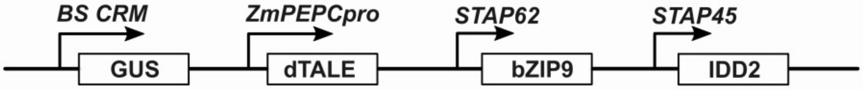
BS CRM
ZmPEPCpro
STAP62
STAP45
GUS
dTALE
bZIP9
IDD2

h

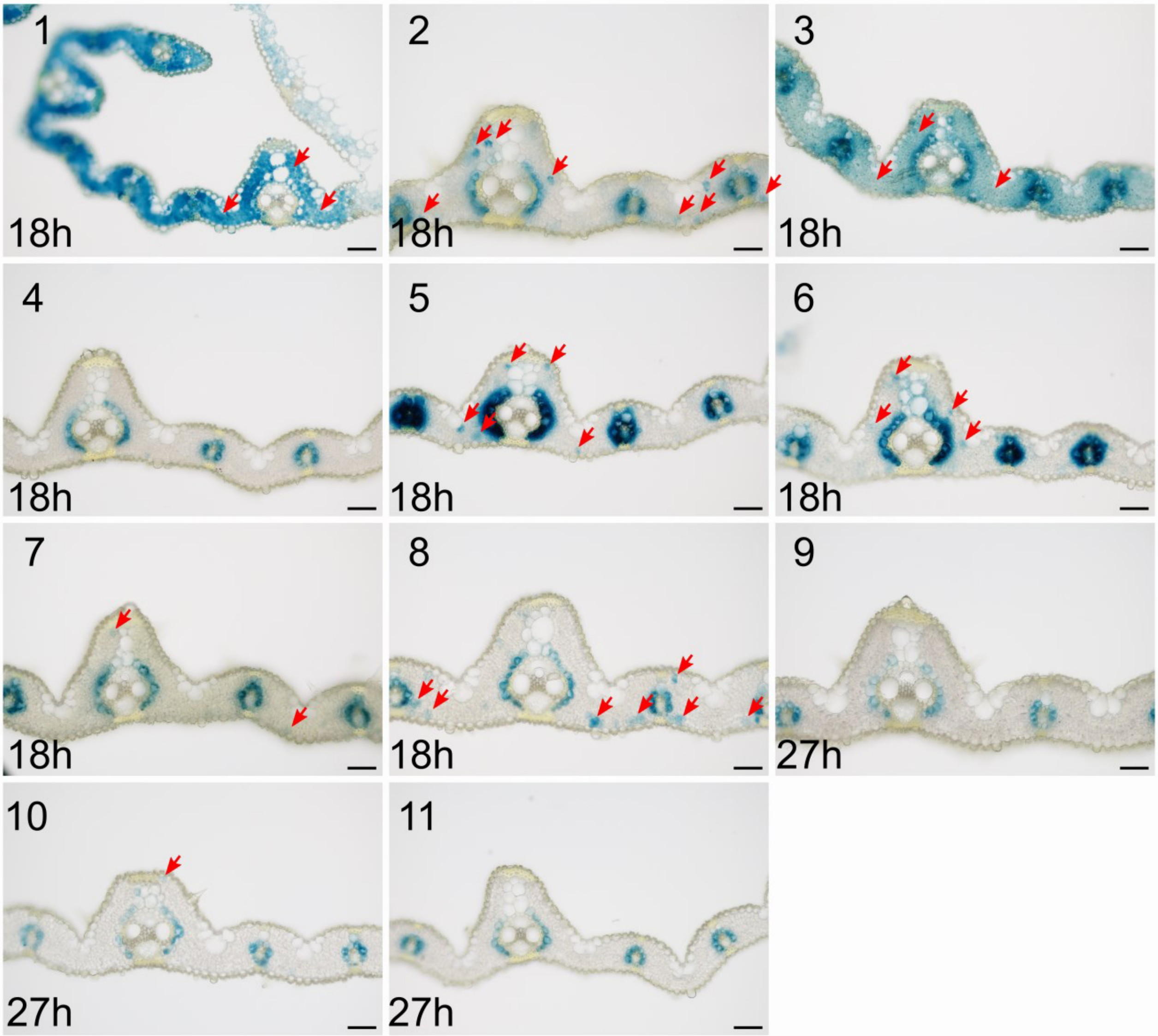
1
18h
2
18h
3
18h
4
18h
5
18h
6
18h
7
18h
8
18h
9
27h
10
27h
11
27h

i

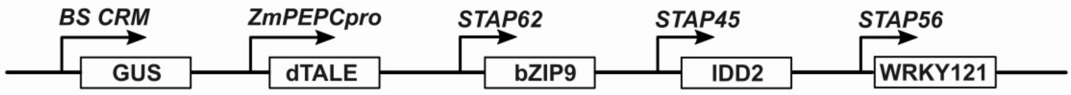

j

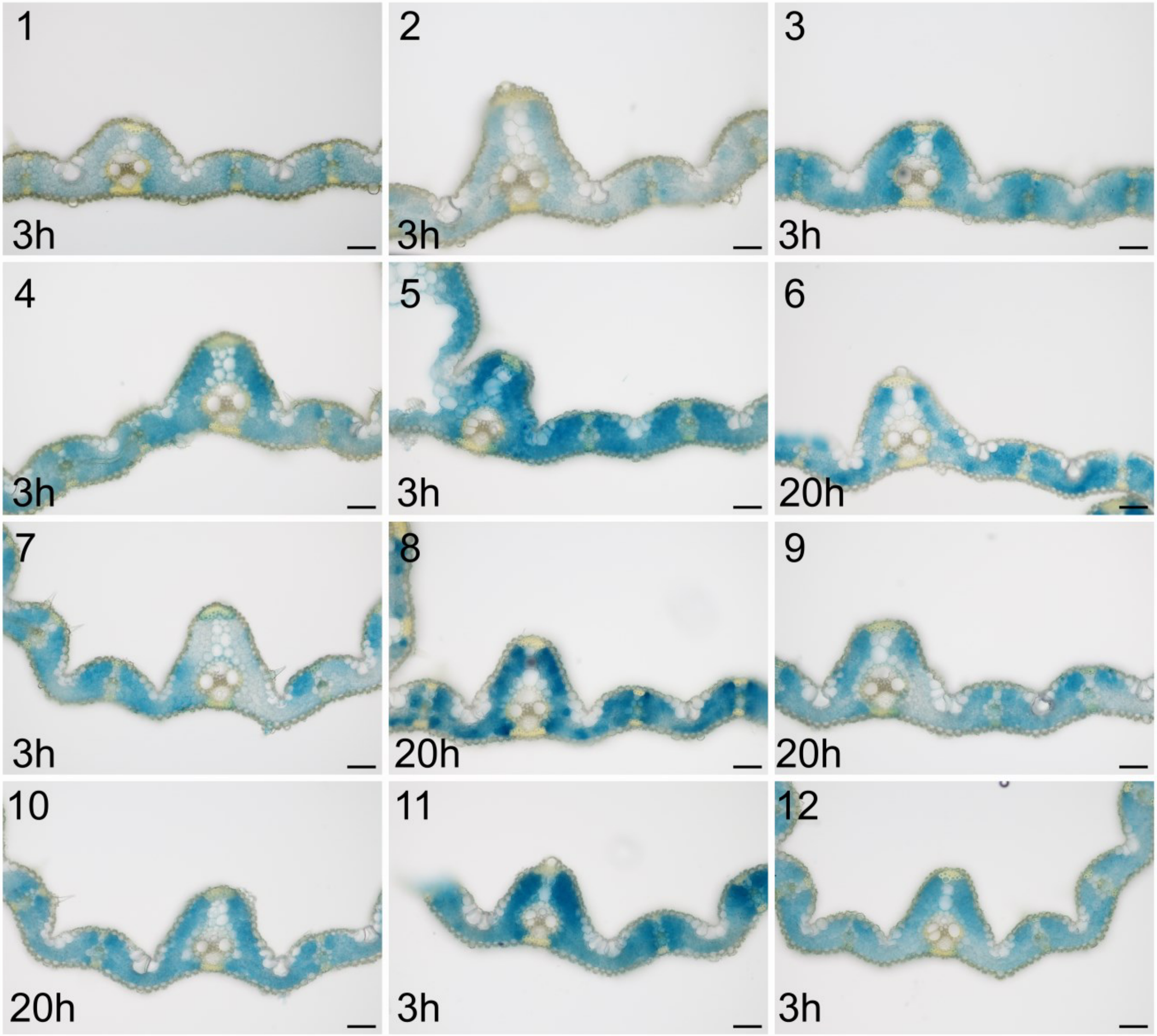

Supplementary Figure 15. Impact of mis-expression of bZIP9, IDD2 and WRKY121 in mesophyll cells on GUS expression pattern driven by the bundle sheath CRM. (**a,c,e,g,i**) Schematics showing mis-expression of bZIP9, IDD2 and WRKY121 in the mesophyll cells using the maize *PEPC* promoter driving dTALE in combination with *STAP56*, *45* and *62* or maize *UBI* promoter (IDD2), the bundle sheath CRM was fused with the *SiR* core promoter and GUS gene as a reporter. (**b,d,f,h,j**) Representative images of cross sections from transgenic lines after GUS staining, scale bars = 50 µm. Nine, six, nine, eleven and twelve independent transgenic lines shown for each construct. Red arrows indicate GUS expressing mesophyll cells. Staining duration is displayed in the bottom-left corner, scale bars = 50 µm in **b,d,f,h,j**.

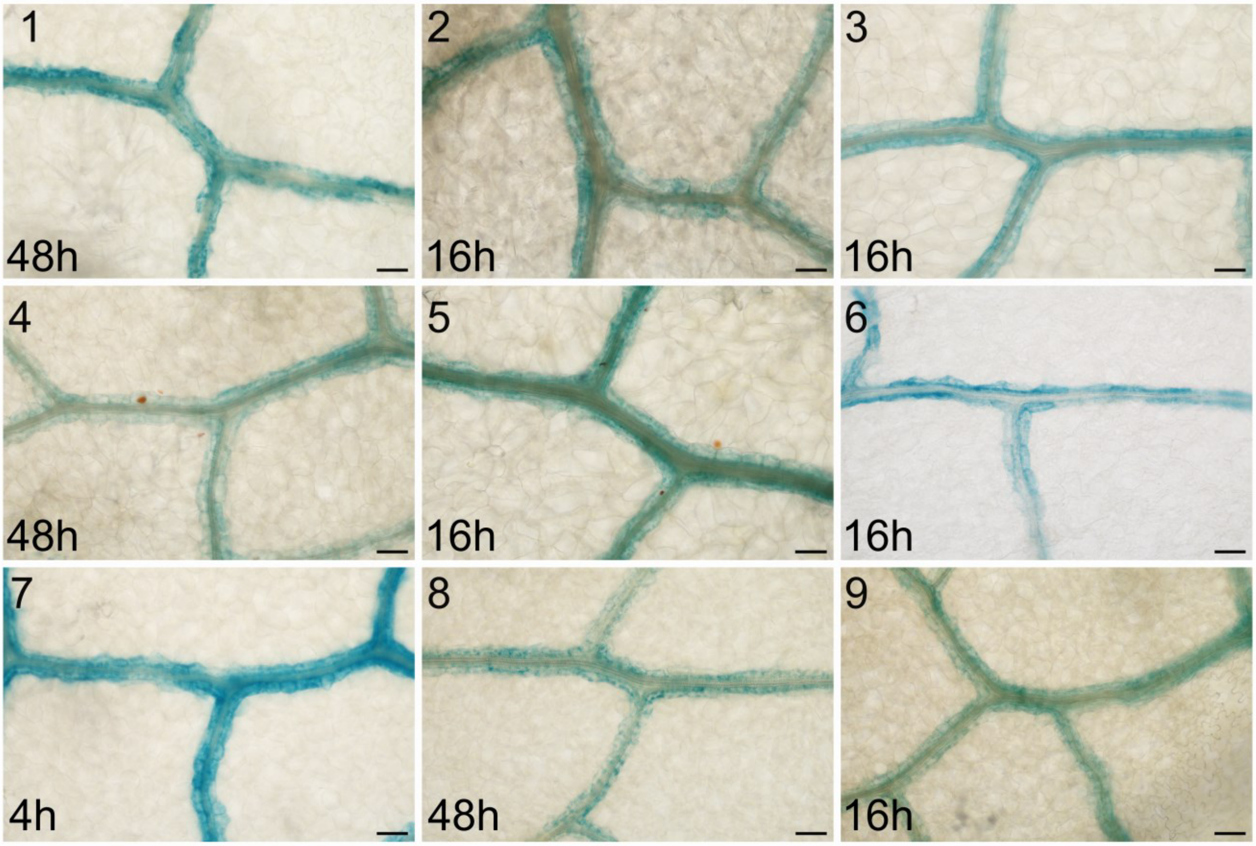

Supplementary Figure 16. Three copies of the bundle sheath CRM produce strong and specific bundle sheath specific expression in Arabidopsis. Nine independent lines shown. Staining duration displayed in the bottom-left corner of each image, scale bars = 50 μm.

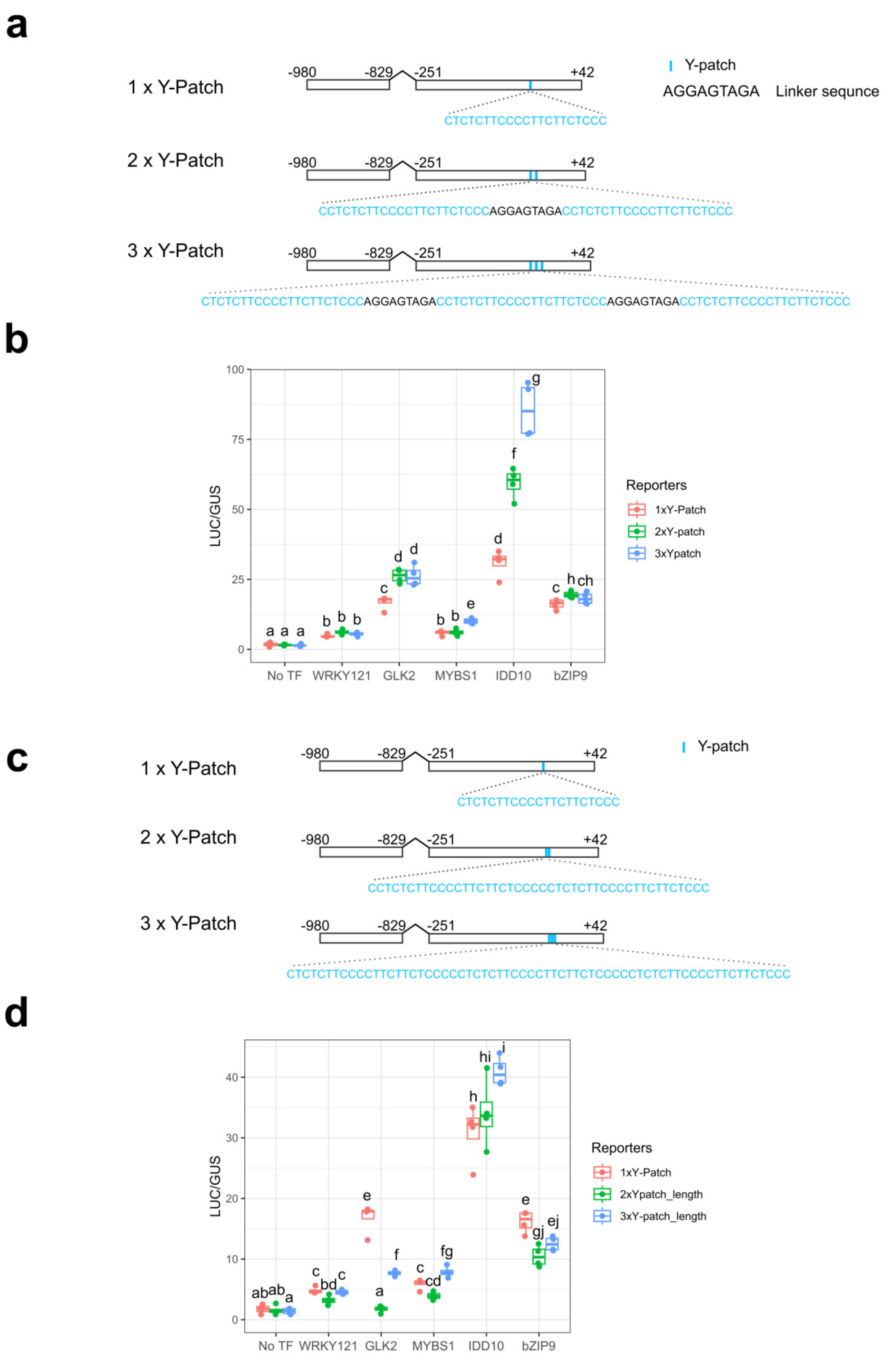
a
1 x Y-Patch
-980
-829
-251
+42
CTCTCTTCCCCTTCTTCTCCC
Y-patch
AGGAGTAGA Linker sequnce
2 x Y-Patch
CCTCTCTTCCCCTTCTTCTCCCAGGAGTAGACCTCTCTTCCCCTTCTTCTCCC
3 x Y-Patch
CTCTCTTCCCCTTCTTCTCCCAGGAGTAGACCTCTCTTCCCCTTCTTCTCCCAGGAGTAGACCTCTCTTCCCCTTCTTCTCCC
b
LUC/GUS
100
75
50
25
0
No TF
WRKY121
GLK2
MYBS1
IDD10
bZIP9
Reporters
1xY-Patch
2xY-patch
3xYpatch
c
1 x Y-Patch
-980
-829
-251
+42
CTCTCTTCCCCTTCTTCTCCC
Y-patch
2 x Y-Patch
CCTCTCTTCCCCTTCTTCTCCCCCTCTCTTCCCCTTCTTCTCCC
3 x Y-Patch
CTCTCTTCCCCTTCTTCTCCCCCTCTCTTCCCCTTCTTCTCCCCCTCTCTTCCCCTTCTTCTCCC
d
LUC/GUS
40
30
20
10
0
No TF
WRKY121
GLK2
MYBS1
IDD10
bZIP9
Reporters
1xY-Patch
2xYpatch_length
3xY-patch_length

Supplementary Figure 17. Two or three copies of Y-patch but not longer Y-patch enhances the transcriptional activity from the bundle sheath CRM. (**a**) Schematic showing reporter sequences used in the transactivation assay with two or three copies of Y-patch separated by 10-bp linker sequence, the original core promoter was used as 1xY-patch control. (**b**) effector assay showing transcriptional activation is enhanced by GLK2, MYBS1, IDD10 and bZIP9 using two or three copies of Y-patches compared with the control reporter. (**c**) Schematic showing reporter sequences with 40-bp and 60-bp Y-patch, the original core promoter was used as 20-bp Y-patch control. (**d**) Effector assay showing transcription activity was unchanged or repressed using 40 or 60-bp Y-patch compared with the 20-bp Y-patch control. In **b**&**d**, Log_2 transformed LUC/GUS ratio is subjected to pairwise t-test with Benjamini-Hochberg correction, box plots display the median and the 25th, 50th and 75th percentiles; whiskers extend to the furthest data points within 1.5 times the interquartile range, samples with significantly different activities (adjusted *P*<0.05) are labelled with different letters. Source data are provided as a Source Data file.

	with the CRM and Y-patch	without the CRM or Y-patch	Total
Number of genes	282	41907	42189
Expressed in BS (TPM>=5)	117	7483	7590
Preferentially expressed in BS or BSV clusters (TPM>5)	21	1050	1071
Poorly expressed in BS (TPM<5) (not expressed in leaf)	73 (93)	7505 (26928)	7578 (27021)
Ratio of expressed genes TPM>5 in genome	0.415	0.179	Odd ratio=3.26, $P < 2.2e\text{-}16$
Ratio of BS-preferential genes in genome	0.074	0.025	Odd ratio=3.13, $P = 1.304e\text{-}05$

Supplementary Figure 18. Genome wide summary of CRM and Y-patch prevalence in rice. Fisher's exact test demonstrates that the bundle sheath CRM and Y-Patch are overrepresented in DNase I Hypersensitive Sites associated genes expressed in the rice bundle sheath. Core promoters were assessed for presence of a Y-patch, and this was combined with identification of DNase I Hypersensitive Sites in both promoter and gene bodies. Presence of both the bundle sheath CRM and the Y-patch is significantly overrepresented in genes expressed in the bundle sheath, but also those preferential to the bundle sheath. The remaining 73 genes were poorly expressed in the bundle sheath and 93 were not detectable in leaves. Data from Hua et al., 2021[1].

References

1 Hua, L. *et al.* The bundle sheath of rice is conditioned to play an active role in water transport as well as sulfur assimilation and jasmonic acid synthesis. *The Plant Journal* **107**, 268–286 (2021).

2 Picot, E., Krusche, P., Tiskin, A., Carré, I. & Ott, S. Evolutionary analysis of regulatory sequences (EARS) in plants. *The Plant Journal* **64**, 165–176 (2010).

3 Zhang, Y. *et al.* Rice co-expression network analysis identifies gene modules associated with agronomic traits. *Plant Physiol* **190**, 1526–1542 (2022).

4 Fornes, O. *et al.* JASPAR 2020: Update of the open-Access database of transcription factor binding profiles. *Nucleic Acids Res* **48**, D87–D92 (2020).
