# Supplementary material for: A transcription factor ensemble orchestrates bundle sheath expression in rice
Source: Nat Commun. 2025 Jul 31;16:7040. doi: 10.1038/s41467-025-62087-0 (PMC12314071; doi:10.1038/s41467-025-62087-0)
Supplement: Supplementary file 2 — Description of Additional Supplementary Files [file 41467_2025_62087_MOESM2_ESM.pdf]

Description of Additional Supplementary Files

Title: Supplementary Data 1

Description: Predicted transcription factor binding sites in rice *SiR* promoter using FIMO

Title: Supplementary Data 2

Description: Motif clusters and transcription factor families

Title: Supplementary Data 3

Description: IR64 BSS M LCM RNAseq data

Title: Supplementary Data 4

Description: Bundle sheath preferential genes identified from Hua *et al.*, 2021

Title: Supplementary Data 5

Description: Leaf developmental gradient LCM RNAseq data

Title: Supplementary Data 6

Description: Transcription factors preferentially expressed in bundle sheath strands during leaf maturing

Title: Supplementary Data 7

Description: Primer sequences of promoters

Title: Supplementary Data 8

Description: Primer sequences used for *SiR* promoter domestication and core promoter cloning

Title: Supplementary Data 9

Description: Primer sequences used for cloning transcription factors

Title: Supplementary Data 10

Description: List of genes containing the bundle sheath CRM and Y-patch in rice genome
